# The predictors of lymphopenia and its effects on survival in locally advanced esophageal squamous cell carcinoma

**DOI:** 10.1080/15384047.2024.2371632

**Published:** 2024-07-01

**Authors:** Danjing Luo, Qiulu Zhong, Haiying Yue, Jue Wang, Qianfu Liang, Wenqi Liu, Xiaodong Zhu

**Affiliations:** a Department of Radiation Oncology, Second Affiliated Hospital of Guangxi Medical University, Nanning, China; b Department of Radiation Oncology, Guangxi Medical University Cancer Hospital, Nanning, China; c Department of Oncology, Wuming Hospital of Guangxi Medical University, Nanning, China

**Keywords:** Lymphopenia, the effective radiation dose to immune cells, esophageal squamous cell carcinoma, definitive radiation therapy, gross tumor volume, survival

## Abstract

To investigate the impact of the effective radiation dose to immune cells (EDIC) and gross tumor volume (GTV) on lymphopenia and survival in patients with locally advanced esophageal squamous cell carcinoma (LAESCC). Between January 2013 and December 2020, 272 LAESCC patients were treated with definitive radiotherapy in two institutions. Based on radiation doses to the lungs, heart, and body region scanned, EDIC was calculated as an equal uniform dose to the total blood considering blood flow and fraction effect. The radiotherapy plan was used to calculate the GTVs. Lymphopenia was graded based on the lowest lymphocyte count during RT. The overall survival (OS), progress-free survival (PFS), and local recurrence-free survival (LRFS) were analyzed statistically. The lowest lymphocyte count was significantly correlated with EDIC (*r*= −0.389, *p* < .001) and GTV (*r*= −0.211, *p* < .001). Lymphopenia, EDIC, and GTV are risk factors for patients with ESCC. In a Kaplan-Meier analysis with EDIC and GTV as stratification factors, lymphopenia was not associated with OS in the EDIC>12.9 Gy group (*p* = .294)and EDIC ≤ 12.9 Gy group, and it was also not associated with OS in GTV>68.8 cm^3^ group (*p* = .242) and GTV ≤ 68.8 cm^3^ group(*p* = .165). GTV and EDIC had an impact on the relationship between lymphopenia and OS in patients with LAESCC undergoing definitive RT. Poorer OS, PFS, and LRFS are correlated with lymphopenia, higher EDIC, and larger GTV.

## Introduction

The immune system is essential for preventing and curing cancer,^1,2^ and peripheral blood lymphocytes are important immune system constituents.^3,4^ Previous studies have discovered a relationship between radiation-related lymphopenia with poorer survival of various cancers, including esophageal carcinoma (ECC).^5–10^ Therefore, finding impact factors of lymphopenia can help to improve efficacy and choose high-risk ECC patients for treatment optimization and follow-up management.

However, most previous studies concentrated on adenocarcinoma and included patients at various stages of the disease. Furthermore, the lymphopenia cutoff values varied across studies. These factors may have influenced the outcome. As a result, we conducted this study to investigate the lymphopenia criteria and see if GTV and EDIC can influence lymphopenia and poor outcomes in patients with locally advanced esophageal squamous cell carcinoma (LAESCC).

## Materials and methods

### Patients

Patients treated with definitive (chemo) radiotherapy (RT) at the radiation oncology department of the Second Affiliated Hospital and the Cancer Hospital of Guangxi Medical University between January 2013 and December 2020 were selected. 272 patients were included with stage II-IVA (American Joint Committee on Cancer, eighth edition, cM1, positive nonregional lymph nodes and irradiated during radiotherapy).

### Treatment

Patients received a total radiation dose of 50 to 70.4 Gy in 25–33 fractions with elective nodal irradiation. Radiation was administered for 5–7 weeks using IMRT. All patients received definitive RT with or without chemotherapy. The chemotherapy regimens were nedaplatin/cisplatin and paclitaxel/docetaxel/5-fluorouracil.

### Data collection

The following information was gathered from medical records: age, gender, performance status, smoking history, drinking history, heart disease history, pathology, staging, differentiation, tumor location, treatment modality, and lymphocyte count. And the RT plan provided data for radiation dose, gross tumor volume (GTV), mean lung dose (MLD), mean heart dose (MHD), and mean body dose (MBD).

Lymphocyte counts (cells × 10^9^/L) were collected 1 week before the start of treatment, every week during RT, and 2 weeks after RT. Lymphopenia was graded based on the lowest lymphocyte count and the Common Terminology Criteria for Adverse Events, version 5.0. G4 nadir was defined as lymphocyte count<0.2 × 10^9^/L.

The effective radiation dose to immune cells (EDIC) was calculated as follows using the model developed by Jin et al.^11^and improved by Ladbury et al.^12^:

$$\mathrm{EDIC}=0.12\times \mathrm{MLD}+0.08\times \mathrm{MHD}+\left[0.45+0.35\times 0.85{\left(\frac{\#\mathrm{of}\;\mathrm{fraction}}{45}\right)}^{\frac{1}{2}}\right]\times \mathrm{MBD}$$

### Statistical analysis

The primary endpoint of this study was Overall survival (OS) and the secondary endpoint was progression-free survival (PFS) and locoregional recurrence-free survival (LRFS). The survival duration was calculated from the start of treatment to the corresponding event (loco-regional recurrence, tumor progression, death, or the last follow-up).

To compare continuous and categorical variables, the independent sample t-test or the χ^2^ test was used. According to receiving operating characteristic (ROC) survival curve analysis, the best cutoff value for EDIC and GTV was determined. The univariate and multivariate Cox regression models were used to investigate the relationship between survival and variables (inclusion criteria, p < .1). To examine correlations between variables, Spearman’s rank correlation coefficient was utilized. Spearman’s rank correlation coefficient was used to test correlations between variables. The tests were considered statistically significant if *P* < .05. All the *p* values were two-sided. SPSS 22.0 (IBM, Chicago, IL) and GraphPad Prism 9 were used for the statistical analyses.

## Results

A total of 272 patients were analyzed. The patient characteristics are listed in Table 1. The median follow-up was 54 months (2–107), and 73.9% (201/272) died. A median dose of 61.5 Gy (range, 50–70.4) was administered to all patients. The 3-year OS, PFS, and LRFS rates were 24.6%, 21.3%, and 22.9%, respectively, with a median of 15, 12, and 13 months, respectively. According to the ROC curve, the median cutoff point for EDIC was 12.9 Gy (range, 2.4–19.2) and 68.8 cm^3^ (range,4.1–255.4) for GTV. In univariate analysis, lymphopenia, EDIC, GTV, and clinical stage are risk factors for OS, PFS, and LRFS (Table 2). In multivariate analysis, only EDIC and GTV are significant prognostic factors for OS, PFS, and LRFS (Table 3).

**Table 1.** Patient characteristics.

characteristics	Number of Patients (%)
Age,median(range,years)	60(range, 40–84)
≥60	148(50.7)
<60	134(49.3)
Gender	
Male	238(87.5)
Female	34(12.5)
ECOG-score	
0–1	252(92.6)
2	20(7.4)
Smoking history	
Yes	174(64)
No	98(36)
Drinking history	
Yes	183(67.3)
No	89(32.7)
Heart disease history	
Yes	16(5.9)
No	256(94.1)
Clinical Stage	
II	30(11)
III	158(58.1)
IVA	84(30.9)
Differentiation	
Well/Moderate	162(59.6)
Poor	110(40.4)
Tumor location	
Cervical+Upper 1/3	108(39.7)
Middle 1/3+Lower 1/3	164(60.3)
GTV, average volume (cm)^3^	70.7(range, 4.1–255.4)
RT dose,median (range,Gy)	61.5(range,50–70.4)
Chemotherapy	
Concurrent±Sequential	174(64)
Induction±Concurrent	31(11.4)
Sequential	8(2.9)
None	59(21.7)
Lymphopenia	
G0–3	120(44.1)
G4	152(55.9)

Abbreviations: ECOG: Eastern Cooperative Oncology Group; GTV:gross tumor volume; RT: radiation therapy.

**Table 2.** Univariable analysis of clinical and dosimetric variables with outcomes.

Variables	OS		PFS		LRFS	
	HR	*P*	HR	*P*	HR	*P*
Age	1.027 (0.779–1.355)	.850	0.897 (0.680–1.185)	.445	0.981 (0.743–1.294)	.890
Gender	1.407 (0.911–2.157)	.124	0.651 (0.421–1.006)	.**053**	0.681 (0.441–1.053)	.084
ECOG	1.229 (0.921–1.641)	.162	1.152 (0.872–1.522)	.319	1.163 (0.875–1.545)	.299
Smoking history	1.232 (0.919–1.653)	.163	1.277 (0.953–1.713)	.102	1.279 (0.954–1.716)	.10
Drinking history	0.782 (0.577–1.06)	.113	0.734 (0.542–0.995)	.**046**	0.778 (0.574–1.055)	.106
Heart disease history	0.908 (0.517–1.596)	.738	1.144 (0.651–2.01)	.639	1.095 (0.623–1.924)	.753
Clinical Stage	1.367 (1.078–1.733)	.**01**	1.414 (1.118–1.789)	.**004**	1.365 (1.078–1.728)	.**01**
Differentiation	0.856 (0.701–1.046)	.129	0.876 (0.716–1.071)	.195	0.878 (0.718–1.072)	.202
Tumor location	1.097 (0.928–1.297)	.28	1.121 (0.949–1.323)	.179	1.107 (0.937–1.307)	.233
GTV	0.589 (0.444–0.781)	**<.001**	0.537 (0.404–0.713)	**<.001**	0.577 (0.435–0.766)	**<.001**
EDRIC	0.551 (0.417–0.729)	**<.001**	0.521 (0.394–0.690)	**<.001**	0.554 (0.419–0.733)	**<.001**
RT dose	1.166 (0.883–1.540)	.280	1.061 (0.803–1.401)	.676	1.113 (0.843–1.471)	.450
Lymphopenia	1.374 (1.035–1.823)	.**028**	1.408 (1.061–1.868)	.**018**	1.401 (1.055–1.860)	.**020**

**Table 3.** Multivariate analysis of clinical and dosimetric variables with outcomes.

Variables	OS		PFS		LRFS	
	HR	*P*	HR	*P*	HR	*P*
Gender			0.859 (0.504–1.463)	.576		
Drinking history			1.185 (0.820–1.714)	.367		
Clinical Stage	1.094 (0.842–1.422)	.499	1.147 (0.887–1.483)	.295	1.088 (0.839–1.41)	.525
GTV	0.720 (0.528–0.982)	.**038**	0.666 (0.490–0.907)	.01	0.699 (0.513–0.952)	.**023**
EDRIC	0.673 (0.488–0.928)	.**016**	0.676 (0.489–0.937)	.019	0.689 (0.498–0.953)	.**025**
Lymphopenia	1.171 (0.866–1.583)	.306	1.124 (0.828–1.526)	.454	1.195 (0.884–1.616)	.247

### Lymphocyte count

Before RT, the average lymphocyte count of 272 patients was 1.81(0.38–3.81 × 10^9^/L), and after RT, it was 0.21(0.02–0.74 × 10^9^/L)(*p* < 0.001). There were no patients with G4 nadir before RT, but 152 patients with G4 lymphopenia after RT (*p* < .001).

Patients who received concurrent chemotherapy (CRT group, *n* = 186) were compared to those without concurrent chemotherapy (RT group, *n* = 86). Before RT, the average lymphocyte count in the CRT group was 1.85 (0.38–3.58 × 109/L), while in the RT group it was 1.72 (0.53–3.81 × 109/L), with no statistically significant differences (*p* = .097). After RT, the average lymphocyte count in the CRT group was 0.19 (0.02–0.71 × 109/L), while in the RT group it was 0.23 (0.02–0.74 × 109/L), with no statistically significant differences (*p* = .053).

### Lymphopenia and survival

**Figure 1.**
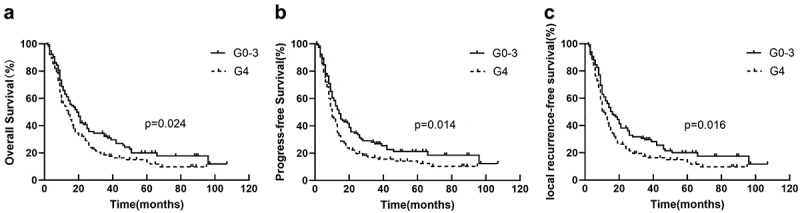
Kaplan-Meier curves for OS(a), PFS(b), and LRFS(c) between patients with the G0–3 group and G4 group.

Compared to G4 nadir, patients with G0–3 nadir had a significantly better OS (19 vs. 14 months, *p* = .024) (Figure 1a), PFS (14 vs. 10 months, *p* = .014) (Figure 1b), and LRFS (16 vs. 11 months, *p* = .016) (Figure 1c).

### Lymphopenia correlate with EDIC result in survival

The lowest lymphocyte count was correlated with EDIC (*r* = −0.389, *p* < .001). To investigate the relationship between lymphopenia and EDIC, the EDIC was divided into quartiles based on prevalence, and the percentage of G4 lymphopenia was 32.4% (2.4–10.3 Gy), 44.1% (10.3–12.7 Gy), 69.1% (12.7–15.3 Gy), and 77.9% (15.3–19.2 Gy), respectively (*p* < .001).

**Figure 2.**
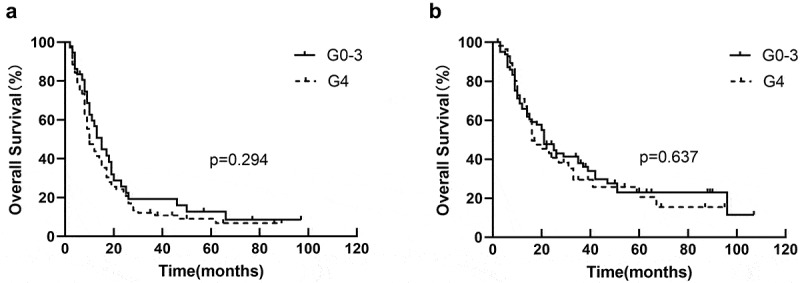
Kaplan-Meier curves for OS by dichotomy (a) in EDRIC>12.9 Gy group, and (b) in EDIC ≤12.9 Gy group.

EDIC was used as a stratification factor in univariate analysis and Kaplan-Meier analysis to further investigate whether EDIC influences the prognostic role of lymphopenia on survival. After considering EDIC as a stratification factor, there was no discernible connection between lymphopenia and OS (HR = 1.184, *p* = .26) in univariate analysis. In Kaplan – Meier analysis, lymphopenia was not associated with OS in the EDIC>12.9 Gy group (*p* = .294) (Figure 2a) and EDIC ≤12.9 Gy group (*p* = .637) (Figure 2b).Other survival statistics (PFS and LRFS) were list in Table 4.

**Table 4.** Kaplan-Meier analysis of lymphopenia with outcomes.

		OS		PFS		LRFS	
Variables	lymphopenia	Median 95%CI(months)	*P*	Median 95%CI(months)	*P*	Median 95%CI(months)	*P*
ALL	G0–3	19 (14.9–23.1)	.024	14 (11.2–16.8)	.014	16 (11.2–20.8)	.016
	G4	14 (11.2–16.8)		10 (8.4–11.6)		11 (9.1–12.9)	
GTV>68.8 cm^3^	G0–3	17 (7.5–26.5)	.242	9 (6.3–11.7)	.305	12 (6.6–17.4)	.18
	G4	10 (9.0–11.0)		8 (6.6–9.4)		9 (7.5–10.5)	
GTV ≤68.8 cm^3^	G0–3	21 (14.2–27.8)	.165	16 (10.5–21.5)	.095	20 (13.9–26.1)	.134
	G4	17 (14.5–19.5)		13 (10.9–15.1)		15 (12.7–17.3)	
EDIC>12.9 Gy	G0–3	15 (10.8–19.2)	.294	10 (7.3–12.7)	.575	11 (7.8–14.2)	.322
	G4	10 (7.3–12.7)		8 (7.0–9.0)		10 (8.8–11.2)	
EDIC ≤12.9 Gy	G0–3	21 (14.9–27.1)	.637	20 (13.2–26.8)	.365	20 (12.3–27.7)	.446
	G4	16 (9.4–22.6)		13 (10.5–15.5)		15 (11.0–19.0)	

### Lymphopenia correlate with GTV result in survival

The lowest lymphocyte count was associated with GTV (*r* = −0.211, *p* < .001). To investigate the relationship between lymphopenia and GTV, the GTV was divided into quartiles based on prevalence, and the percentage of G4 lymphopenia was 41.2% (4.1–38.4 cm^3^), 51.5% (38.4–57.9 cm^3^), 63.2% (57.9–92.2 cm^3^), and 67.6% (92.2–255.4 cm^3^), respectively (*p* < .001).

**Figure 3.**
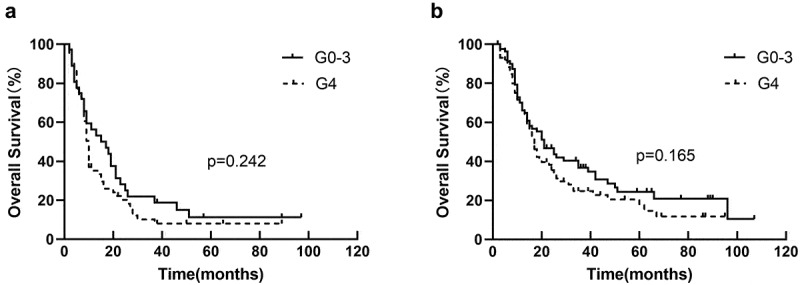
Kaplan-Meier curves for OS by dichotomy (a) in GTV>68.8 cm^3^ group, and (b) in GTV ≤68.8 cm^3^ group.

There was no significant correlation between lymphopenia with OS after using GTV as a stratification factor (HR = 1.321, *p* = .055) in univariate analysis. In Kaplan – Meier analysis, lymphopenia was not associated with OS in GTV>68.8 cm^3^ group (*p* = .242) (Figure 3a) and GTV ≤68.8 cm^3^ group (*p* = .165) (Figure 3b). Other survival statistics (PFS and LRFS) were list in Table 4.

## Discussion

In our study, we found that definitive RT for LAESCC can reduce the lymphocyte count, and whether concurrent chemotherapy has no discernible effect on the lymphocyte count. We also discovered that lymphopenia was linked to poor survival, which was influenced by GTV and EDIC. After adjusting for GTV and EDIC, lymphopenia’s prognostic role during RT remained insignificant. The higher EDIC and larger GTV correlate with worse OS, PFS, and LRFS.

As we all know, RT is an important treatment for ECC.^13–15^ According to growing clinical data, low absolute lymphocyte count (ALC) during RT is associated with poor survival of ECC. A retrospective study^10^ enrolled 504 patients with stage I-III ECC who received neoadjuvant or definitive CRT and discovered that G4 ALC nadir (defined as a nadir of < 200 cells/ml) was correlated with poor OS and disease-specific survival outcomes. The median OS in patients with G0–2 nadir and G4 nadir was significantly different (5.0 vs. 2.8 years, *p* = .027). It should be mentioned that the majority of the included patients were adenocarcinoma and received CRT with 50.4 Gy. To further investigate the role of ALC in patients with esophageal squamous cell carcinoma (ESCC) treated with definitive RT, Wang et al.^16^ conducted a study of 189 patients with ESCC treated with definitive RT (50-68 Gy) combined with or without chemotherapy. Patients were divided into two groups based on the cut-off value of ALC nadir (defined as 0.38 × 10^3^ cells/µl), and there were significant differences in OS (*p* < .001), PFS (*p* = .0048), and LRFS (*p* < .0053) in low ALC nadir group (≤0.38 × 10^3^ cells/µl) and high ALC nadir group(>0.38 × 10^3^ cells/µl). In our study, we also discovered that radiation-related lymphopenia was linked to poor outcomes in patients with LAESCC.

Although the mechanisms underlying the relationship between lymphopenia and the outcome of ECC treatment are still unknown. However, a variety of studies have focused on the identification of risk factors for treatment-related lymphopenia in ECC. Xu et al.^17^ improved the model used to calculate EDIC for ECC patients who received CRT and investigated the relationship between EDIC and lymphopenia. Higher EDIC values (>4 Gy) were associated with G4 nadir during CRT (67.3% vs. 40.8%, *p* < .001), and EDIC score was a significant risk factor for severe lymphopenia. EDIC was also an independent prognostic factor for OS, PFS, and distant metastasis-free survival (DMFS). Another study draws the same conclusion. Cai et al.^18^ found that the cutoff value of EDIC was 10.3 Gy according to the ROC curve, then 146 patients with ECC were divided into the EDIC ≥10.3 Gy group and EDIC<10.3 Gy group, and the final result showed that patients with EDIC ≥10.3 Gy had a significantly lower median OS (14.2 vs.39.6 months, *p* < .001), and PFS (28.1 vs.39.4 months, *p* = .024). It should be noted that they may disregard the impact of the other dosimetry parameters. There are certainly other studies focusing on this issue. Wang et al.^16^ concluded that large PTVs affected a low ALC nadir during RT in the study mentioned above. In addition to PTVs, Xu et al.^19^ showed that G4 nadir was related to other dosimetry parameters such as lung V10 and heart V10 in patients with ESCC treated with definitive CRT. And G0–3 nadir group was significantly associated with better OS (*p* < .001), PFS (*p* < .001), and DMFS (*p* = .008) than the G4 nadir group, but not with locoregional failure-free survival (LRFFS) (*p* = .202). Moreover, to look into the relationship between lymphopenia and radiation dose to marrow during CRT for ECC. Anderson et al.^20^ calculated thoracic vertebra volume spared 5–40 Gy (TVS5–40) and found that TVS10-TVS40 was significantly associated with higher lymphocyte nadirs in 46 patients with ECC. Furthermore, Newman et al.^21^ investigated the relationship between lymphopenia and vertebra volume in 54 patients with ECC, finding that absolute vertebral volume receiving 10 Gy > 289 cc, 20 Gy ≥ 270 cc, and 30 Gy ≥ 197 cc all correlated with absolute lymphocyte nadir. More samples may be required to support this conclusion. Besides that, mixed pathology and stages may have an impact on the results. In our study, we included 272 LAESCC patients and discovered that lymphopenia does not correlate with OS if we used GTV or EDIC as a stratification factor based on the GTV and EDIC cutoff values. Then we concluded that GTV and EDIC affect the relationship between lymphopenia and OS.

Based on our findings, we can propose specific strategies to improve the outcomes. To begin, radiotherapy techniques can be improved to reduce the radiation dose delivered to bone marrow, circulating lymphocyte cells, the lung, and the heart. In addition, we can also identify high-risk patients and modify their treatment, such as induction chemotherapy for GTV reduction. Our study also has several limitations. At first, this was a retrospective study with selective bias. Second, there were limitations in the EDIC model. Third, other factors may correlate with lymphocyte counts, such as infections or medications.

## Conclusions

In conclusion, GTV and EDIC had an impact on the relationship between lymphopenia and OS in patients with LAESCC undergoing definitive RT. Poorer OS, PFS, and LRFS are correlated with lymphopenia, higher EDIC, and larger GTV.’

## Data Availability

All data generated or analyzed during this study are included in this published article.

## Abbreviations

EDIC: The effective radiation dose to immune cells
GTV: Gross tumor volume
PTV: Planning target volume;
MLD: mean lung dose (MLD)
MHD: mean heart dose (MHD);
MBD: mean body dose (MBD)
LAESCC: Locally advanced esophageal squamous cell carcinoma
ECC: Esophageal carcinoma;
OS: The overall survival
PFS: progress-free survival
LRFS: Local recurrence-free survival
DMFS: distant metastasis-free survival
LRFFS: Locoregional failure-free survival
CI: Confidence interval;
CRT: Concurrent chemoradiotherapy
RT: Radiotherapy
IMRT: Intensity-modulated radiotherapy
ROC: receiving operating characteristic
ALC: Absolute lymphocyte count
TVS: Thoracic vertebra volume spared.
